# Serum Thyroglobulin: Preoperative Levels and Factors Affecting Postoperative Optimal Timing following Total Thyroidectomy

**DOI:** 10.1155/2019/1384651

**Published:** 2019-02-20

**Authors:** Anery Patel, Valerie Shostrom, Kelly Treude, William Lydiatt, Russell Smith, Whitney Goldner

**Affiliations:** ^1^Department of Internal Medicine, Division of Diabetes, Endocrine and Metabolism, University of Nebraska Medical Center, Omaha, NE, USA; ^2^College of Public Health, Department of Biostatistics, University of Nebraska Medical Center, Omaha, NE, USA; ^3^AbbVie, Syneos Health, Omaha, NE, USA; ^4^Department of Surgery, Nebraska Methodist Health System and Creighton University, Omaha, NE, USA; ^5^Department of Surgery-Head and Neck Surgical Oncology, Baptist MD Anderson Cancer Center, Jacksonville, FL, USA

## Abstract

**Objective:**

We evaluated if preoperative TG levels affected postoperative levels and if other factors may influence the optimal time to check postoperative TG.

**Methods:**

This is a prospective, observational pilot study. We approved and enrolled 50 subjects ≥ 19 years scheduled for total thyroidectomy and measured serum TG, thyroglobulin antibody (TG ab), and TSH preoperatively and post thyroidectomy at 7-14 days, 4 and 6 weeks, and 3 months in subjects with benign pathology, with additional 6- and 12-month measurements in subjects with thyroid cancer.

**Results:**

Preoperative TG was significantly higher in the benign (median 167.5 ng/mL vs 30.8 ng/mL) than in the malignant (*p* = 0.0006) group. In the benign group, 76.5% (13/17) of subjects had an undetectable TG < 0.2 ng/mL by 12 weeks postoperatively. In the malignant group, 70.6% (12/17) of those who did not receive RAI therapy and 25% (1/4) of those who did receive RAI had undetectable TG < 0.2 ng/mL by 12 weeks. Subset analysis showed 94.1% (16/17) of the benign, 70.6% of the malignant without RAI, and 50% (2/4) of the malignant with RAI achieved TG < 1.0 ng/mL by 6 weeks postoperatively. Four subjects in the malignant group reached undetectable TG levels as early as 7-14 days postoperatively.

**Conclusion:**

Preoperative TG levels did not predict the risk of malignancy nor time to TG nadir postoperatively. We did not find a difference in TG elimination half-life between the benign and malignant groups. The median time to reach undetectable TG levels in both benign and malignant groups who did not receive RAI therapy was 12 weeks. However, those with preexisting hypothyroidism and hyperthyroidism had lower levels of TG overall in the malignant group which can be taken into consideration besides other known factors that can affect TG levels post thyroidectomy. This trial is registered with Clinicaltrials.gov NCT02347683.

## 1. Introduction

Thyroglobulin (TG) is a dimeric glycoprotein (660 kDa) synthesized and stored in the follicular cells of normal thyroid tissue and regulated by Thyroid-Stimulating Hormone (TSH) [1]. Serum TG correlates with the overall volume of thyroid tissue [2] and it is estimated that 1 ng/mL of TG is equivalent to 1 g of thyroid mass, and the serum TG in a person with a normal gland is approximately 20-25 ng/mL [3]. Serum TG levels are expected to be low following total thyroidectomy; hence, serum TG is used as a tumor marker postoperatively in the follow-up of well-differentiated thyroid cancer (DTC) [4]. When evaluating the optimal postoperative level, most studies have used a functional sensitivity of TG < 1 ng/mL (TSH suppressed or stimulated) when determining evidence of recurrence or remission. Postoperative levels of TG > 1-2 ng/mL may indicate possible residual thyroid remnant or persistence of disease [5]. According to the American Thyroid Association (ATA) guidelines [4], postoperative serum TG (on thyroid hormone therapy or after TSH stimulation) can help in assessing the persistence of disease or thyroid remnant and in predicting potential disease recurrence, is useful in decisions regarding additional radioactive iodine (RAI) I-131 therapy, and quantifies response to therapy. The predictive value of the postoperative TG, however, can be significantly influenced by a wide variety of factors including the amount of residual thyroid cancer and/or normal thyroid tissue, TSH level at the time of TG measurement, functional sensitivity of the TG assay, TG cutoff levels used for analysis, individual risk of having radioiodine avid locoregional or distant metastasis, timing and dose of RAI therapy, and time elapsed since total thyroidectomy [6, 7]. The National Comprehensive Cancer Network (NCN) guidelines recommend checking TG 6-12 weeks post thyroidectomy [8], while ATA guidelines state TG should reach its nadir by 3-4 weeks postoperatively in most patients and that the optimal time to check postoperatively is unknown [4]. The half-life of TG is reported to be 1-3 days (or 65 hours) [1, 6], and the current guidelines are based on estimated clearance of TG from circulation after total thyroidectomy. Obtaining the serum TG level too early following total thyroidectomy may not accurately predict the disease status and erroneously suggest a substantial thyroid remnant or residual disease, thereby leading to unnecessary further investigation and aggressive management strategies as well as heightened anxiety on the part of the patient and provider.

Additionally, TG is used in assessing response to therapy for well-differentiated thyroid cancer. A TG of <0.2 ng/mL is consistent with an excellent response to therapy and predicts risk of recurrence of 1-4%. A TG of 0.2-1.0 ng/mL is considered an indeterminate response to therapy and has a predicted risk of recurrence of 15-20%, while a biochemically incomplete response to therapy is a suppressed TG > 1 ng/mL or a stimulated TG > 10 ng/mL with predicted risk of recurrence of 20% [4].

TG, however, is not routinely tested preoperatively, and guidelines do not recommend checking TG as part of the preoperative workup [4]. Many benign conditions can result in increased production of thyroid hormone (Grave's disease or toxic nodules) or increased release of thyroid hormone (thyroiditis) and can be the etiology for higher serum TG levels [2]. Hence, an elevated serum TG in a person with a thyroid nodule does not always indicate thyroid cancer.

TG is clearly an important part of the evaluation, treatment, and long-term follow-up of DTC; however, optimal timing to check the TG and additional factors that may affect it have not been extensively studied. In this study, we evaluated whether preoperative TG predicted postoperative TG. Additionally, we evaluated other factors that may influence the optimal time to check postoperative TG.

## 2. Methods

This is a prospective observational pilot study. Local IRB approval was obtained. We recruited 50 consecutive adult (age ≥ 19 years) subjects who were planning to undergo total thyroidectomy from March 2015 through February 2016. Subjects consented preoperatively and baseline TG, TG antibody (TG ab), and TSH were obtained prior to surgery. Postoperatively, they were divided into two groups (benign and malignant) based on final pathology. All patients underwent total thyroidectomy with the same group of head and neck surgeons. Subjects were excluded if they were pregnant or lactating. Those with benign pathology were considered the nonmalignant control. They were compared to those with malignant pathology to determine if there was a difference in clearance of TG in the benign and malignant groups. Postoperatively, blood was obtained at predetermined intervals for both the benign and malignant groups. The benign group had TG, TG ab, and TSH at 7-14 days, 4 weeks, 6 weeks, and 3 months postoperatively. The malignant group had TG, TG ab, and TSH at 7-14 days, 4 weeks, 6 weeks, 3 months, 6 months, and 12 months postoperatively. If they had positive TG ab which persisted postoperatively, subjects were excluded since TG ab renders the TG uninterpretable. At our institution, it is standard of care to follow up subjects with thyroid cancer with postoperative ultrasound between 3 and 6 months to monitor any residual or recurrence.

All serum TG and TG Abs were assessed using the Siemens Immulite 1 and were performed at the University of Nebraska Medical Center. The Siemens Immulite 1 is a solid-phase enzyme-labelled, chemiluminescent sequential immunometric assay with a functional sensitivity for TG < 0.2 ng/mL and TG ab < 20 IU/mL. Ultrasensitive TSH assay was done using Beckman Coulter DxI 800 with a reference range of 0.4-5.0 mcIU/mL.

### 2.1. Statistical Analysis

PC SAS version 9.4 was used for all analyses. A time to event analysis using the LIFETEST procedure in SAS was used where the nadir is defined as the event of interest. Time to nadir was compared for subjects with benign pathology versus those with thyroid cancer. Other variables that were compared for those in the benign group versus those in the cancer group include preoperative TG, stage, age (<45 versus ≥45 years), serum creatinine, gender, race, RAI treatment, and histologic type. Chi-square and Fisher's exact tests were used for the categorical variables, and the nonparametric Wilcoxon test was used to compare the continuous variables. Descriptive statistics are provided for all variables. Scatterplots of TSH and TG and bar charts representing the percentage of subjects who reached TG nadir at different time points are provided. We calculated the sample size using a one-sided log-rank test which showed that if 294 subjects are enrolled, that will achieve 80% power at *p* < 0.05 significance level and correspond to a hazard ratio of 0.6986. These results are based on the assumption that the hazard rates are proportional. For our study, we enrolled 50 subjects since it is a pilot study.

## 3. Results

**Figure 1:**
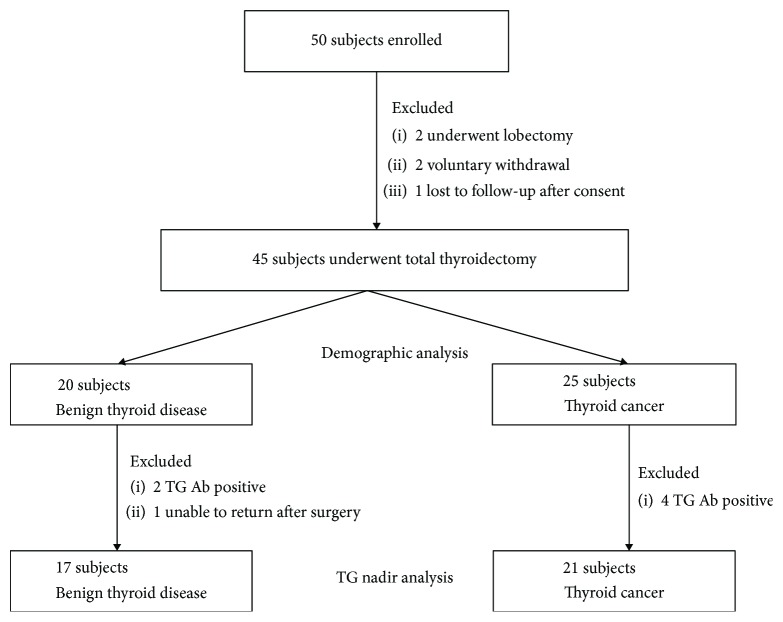
Study enrollment.

Fifty subjects who were planning to undergo total thyroidectomy signed consent. Complete data analysis was performed on 45/50 subjects enrolled (Figure 1). Two subjects were excluded because they underwent lobectomy rather than thyroidectomy after signing consent. Two subjects withdrew because they did not want to return for frequent blood draws, and one subject was lost to follow-up after signing consent and prior to surgery.

Twenty subjects had benign pathology and 25 subjects had thyroid cancer. Seventy-five percent (15/20) of the benign and 72% (18/25) of the malignant group were female (*p* = 0.82). Median age was 52.9 years in the benign and 50.7 years in the malignant group (*p* = 0.65). Sixty percent (15/25) of the malignant histologic diagnoses was classic papillary, 28% (7/25) was follicular variant papillary thyroid cancer (FVPTC), and 12% (3/25) was encapsulated variant FVPTC (NIFTP). Since the study occurred prior to NIFTP classification, NIFTP was included in the malignant group. The majority, 64% (16/25), were categorized as AJCC 7th Edition, stage 1, 16% (4/25) stage 2, 16% (4/25) stage 3, and 4% (1/25) stage 4. When evaluating ATA risk stratification, 64% (16/25) had a low risk, 32% (8/25) had an intermediate risk, and 4% (1/25) had a high risk of recurrence. RAI therapy was administered to 31.8% (7/25) of the subjects. At the conclusion of the study, 68% (17/25) of subjects had an excellent response to therapy, 28% (7/25) had an indeterminate response, and 4% (1/25) had a biochemically incomplete response. None of the subjects had structurally incomplete response to therapy (Table 1).

Preoperative TG was obtained on all subjects at the time of enrollment. The median (range) preoperative TG was 167.5 ng/mL (30.2-2349 ng/mL) in the benign group and 30.8 ng/mL (0.4-264 ng/mL) in the cancer group (*p* = 0.0006) (Table 1).

Of the 45 total subjects, six had persistent positive TG ab postoperatively, so they were excluded from the final analysis. One additional subject had preoperative lab and surgery data available but was unable to return for follow-up after surgery. Overall, 38 subjects were evaluated in the final postoperative TG analysis (Figure 1).

In the malignant group, seven subjects received RAI therapy 6-12 weeks postoperatively. These subjects were evaluated as a subgroup of the malignant group. Overall, TG prior to RAI therapy ranged from 0.2 to 2.0 ng/mL. Three subjects had TG ab postoperatively, making the TG uninterpretable, so they were excluded. All the subjects who underwent RAI therapy received only one dose of I-131 ranging from 100 to 180 mCi.

**Figure 2:**
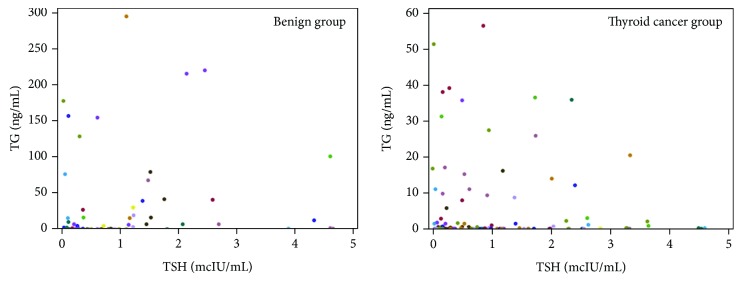
Relation of TSH and TG levels in both groups.

Serum TSH was measured at each time point with TG and TG ab. The relationship of TSH and TG is shown in Figure 2 which includes preoperative TG levels. For the benign group, all the subjects had TSH in the range of 0.5-5 mcIU/mL (90% of subjects with TSH 0.5-2 mcIU/mL postoperatively). For the malignant group, 84% subjects had TSH in the range of 0.5-2 mcIU/mL. One subject had high TSH levels preoperatively (TSH -112 mcIU/mL) while three subjects had TSH > 2 mcIU/mL postoperatively at the time of TG analysis with TSH ranging 2-5 mcIU/mL and TG levels ranging from undetectable to 0.3 ng/mL. All doses were adjusted per standard of care (Figure 2).

### 3.1. TG Analysis with Postoperative TG Cutoff at <0.2 ng/mL

**Figure 3:**
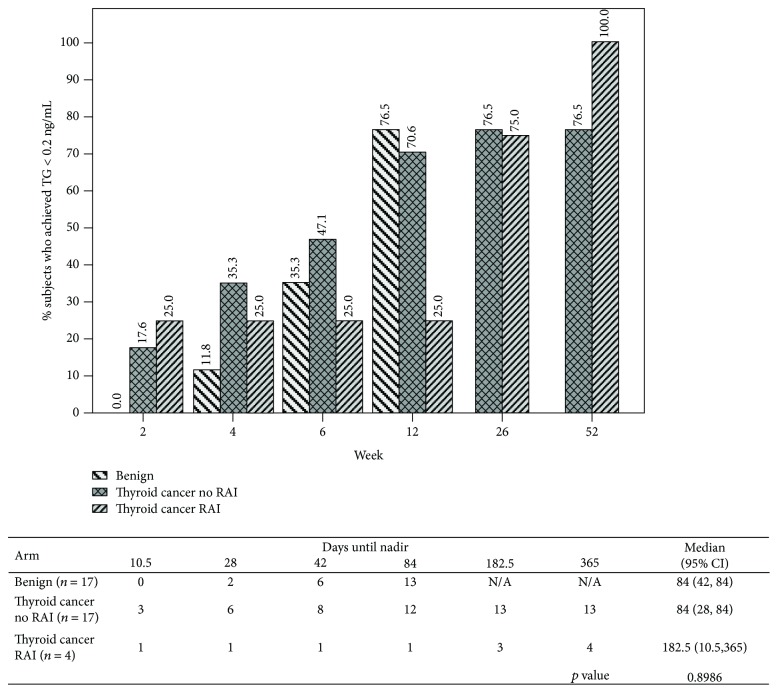
Cumulative number and percentage of subjects who achieved undetectable TG < 0.2 ng/mL at each time point for each group.

In the benign group, 76.5% subjects had undetectable TG < 0.2 ng/mL by 12 weeks postoperatively, while 70.5% of the subjects in the malignant group who did not receive RAI therapy had undetectable TG by 12 weeks. Only 25% of the subjects in the malignant group who received RAI therapy had TG < 0.2 at 12 weeks. Four subjects in the malignant group had undetectable levels as early as 7-14 days postoperatively. The malignant group was followed for one year, and 76.5% percent of subjects who did not receive RAI had undetectable TG by that time. Of the four subjects who received RAI, 100% had undetectable TG levels at one year. Median (95% CI) time required to achieve undetectable TG levels in both benign and malignant groups who did not receive RAI therapy was 12 weeks (benign: 84 days (42, 84 days); malignant: 84 days (28, 84 days)). In subjects who received RAI therapy, median time to achieve undetectable TG was 182.5 days (10.5, 365 days) (*p* = 0.90) (Figure 3).

### 3.2. TG Analysis with Postoperative TG Cutoff at <1.0 ng/mL

**Figure 4:**
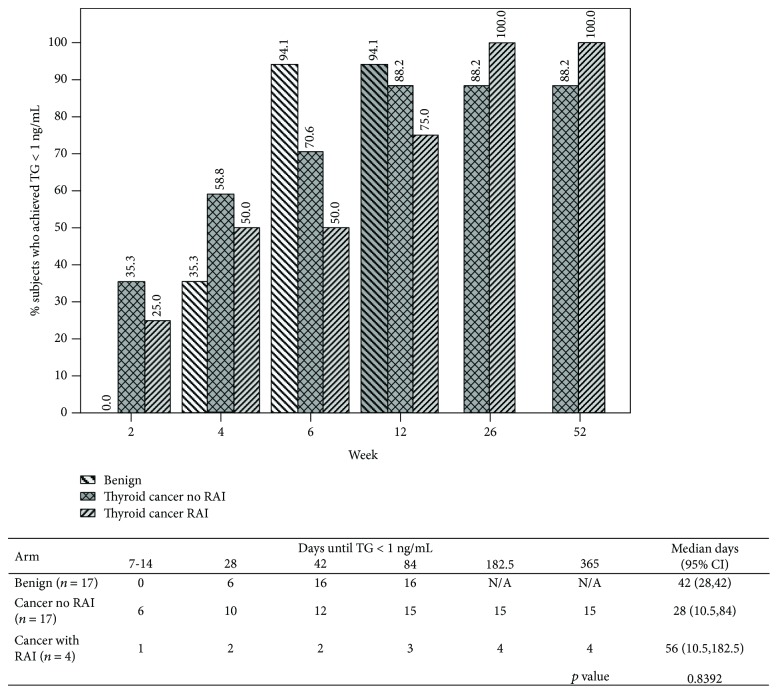
Cumulative number and percentage of subjects who achieved undetectable TG < 1.0 ng/mL at each time point for each group.

We also evaluated time to achieve TG < 1.0 ng/mL rather than undetectable TG. Subset analysis showed 94.1% of the subjects in benign group had TG < 1 ng/mL within 6 weeks after surgery, while 70.6% subjects of the malignant group who did not receive RAI had TG < 1.0 ng/mL by 6 weeks and 50% subjects who received RAI therapy had TG < 1.0 ng/mL by 6 weeks. Median (95% CI) time to achieve TG < 1.0 ng/mL was 6 weeks for the benign group: 42 days (28, 42 days), and 4 weeks for the malignant group who did not receive RAI therapy: 28 days (10.5, 84 days), while it was 8 weeks for the malignant group who received RAI therapy: 56 days (10.5, 182.5 days) (*p* = 0.84) (Figure 4).

### 3.3. TG Analysis in Each Subject

**Figure 5:**
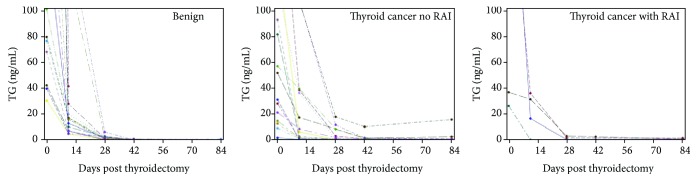
TG level trend in each subject following total thyroidectomy.

Time to achieve TG nadir for each subject in both the groups is shown in Figure 5. In the benign group, 4/17 subjects had detectable TG > 0.2 ng/mL at the conclusion of the study (12 weeks). One subject only completed the 4-week follow-up visit, and the TG was detectable but <1.0 ng/mL. Three subjects had detectable TG ranging from 0.3 to 0.9 ng/mL. In the malignant group, 4/21 subjects had detectable TG > 0.2 ng/mL at the conclusion of the study (1 year). One subject only completed the 4-week labs, and TG was 0.4 ng/mL. One subject had TG of 0.3 ng/mL consistently but with a nonsuppressed TSH of >2 mcIU/mL. Two subjects had a TG > 2.0 ng/mL at the conclusion of the study. One of the subjects had Grave's disease and thyroid cancer, and follow-up ultrasound imaging postoperatively showed residual thyroid tissue but no abnormal masses or lymphadenopathy. The other subjects did not have any thyroid bed tissue or abnormal findings on ultrasound imaging.

## 4. Discussion

Determining the optimal timing of postoperative serum TG measurement is important since it plays an important role in clinical decision making. Guidelines recommend a broad testing window of 3-12 weeks postoperatively; this recommendation is largely based on previous studies of TG elimination half-life. Previous studies evaluating the elimination half-life of TG vary from 6-96 hours and the variation was thought to be due to differences in methodology to measure TG levels. One study reported TG is eliminated through the liver and its half-life following total thyroidectomy was 65.2 hours (range: 36.9-86.6 hours) when measured by RIA to eliminate interassay variations. The TG level was noted to decrease to less than 5-10 ng/mL 25 days after thyroidectomy, or after 7 to 10 half-lives in 11 patient samples [1]. Another study showed that TG with different molecular sizes (ranging 100-600 kDa) was found in the systemic circulation, and following subtotal thyroidectomy, the heaviest TG molecule (19S) has a mean disappearance rate of 4.3 days while the half-life of smaller molecules had a mean disappearance rate value of 3.7 hours [9]. Gerfo et al. suggested that serum TG measurement done at 1 month after total thyroidectomy is indicative of the presence or absence of metastatic/residual disease [10]. Ronga et al. measured serum TG levels 5 weeks after total thyroidectomy for DTC before administering RAI and showed that an elevated serum TG level > 69 ng/mL measured at 5 weeks post total thyroidectomy has a 90% predictive value for the presence of DTC metastases [11]. Lima et al. collected serial weekly samples from 42 patients with DTC for 3 weeks and concluded that serum TG > 2.3 ng/mL at 3 weeks postoperatively can be suggestive of metastasis and need for RAI therapy [12]. In this study, we included patients with benign pathology to compare to those with malignant pathology since previous studies suggested there could be different TG molecular structure according to their cell of origin if produced by benign or malignant cells and hence could take a different amount of time to be eliminated. Our study did not show any significant difference in median time for TG nadir.

In our study, the median time to achieve an undetectable TG level postoperatively in both the benign and malignant groups who did not receive RAI therapy was 12 weeks. However, not surprisingly for those who received RAI therapy, it can take as long as 6 months for undetectable TG levels. If using a TG cutoff of <1.0 ng/mL, median time to goal is 4-8 weeks. This is consistent with the range found in previously published studies [1, 6, 10–12]. It stands to reason that the lower the TG target, the longer one should wait before testing postoperative TG, since TG continues to decline over time. According to our data, waiting until at least 4 weeks and optimally 12 weeks may be more representative of a true TG nadir postoperatively.

By obtaining preoperative TG levels, this allowed us to evaluate the role of preoperative TG levels in predicting the risk of malignancy. We found no correlation between preoperative TG levels and malignancy. In fact, two subjects in the benign group had markedly elevated TG values > 2000 ng/mL. Both of these patients had no evidence of preexisting thyroid disease, had negative TG ab, and had homogenous appearing thyroid parenchyma separate from the bilateral thyroid nodules with indeterminate thyroid FNA biopsies (Bethesda III and IV). The reason for the marked elevation in TG is unclear. This observation supports the ATA recommendation #3 against preoperative TG testing to predict risk of malignancy [4].

Conversely, we had two subjects with thyroid cancer who had preoperative TG levels < 0.2 ng/mL. Both subjects had preexisting hypothyroidism and were on levothyroxine therapy. These two subjects were two of the four subjects that had undetectable TG at 7-14 days postoperatively. Of the two remaining subjects with undetectable TG levels in the malignant group at 7-14 days postoperatively, one had preexisting hyperthyroidism due to toxic multinodular goiter and was on methimazole preoperatively. The potential mechanism for these findings could be that subjects with hypothyroidism on chronic levothyroxine therapy can have small gland volume and low endogenous thyroid hormone and TG production at baseline leading to overall low endogenous TG levels that are eliminated quickly after surgery. Similarly, for subjects with hyperthyroidism, antithyroid therapy also suppresses endogenous TG production potentially allowing for lower baseline levels and more rapid elimination of endogenous TG. These possible mechanisms need to be studied further.

## 5. Conclusion

This is an observational pilot study, limited by small numbers; however, it looked at both pre- and postoperative TG levels in subjects with both benign and malignant pathologies. We did not find a difference in TG elimination half-life between the benign and malignant groups. We also did not show correlation between preoperative TG and malignancy. However, those with preexisting hypothyroid and hyperthyroidism had lower levels of TG overall. The median time to reach undetectable TG levels in both the benign and malignant groups who did not receive RAI therapy was 12 weeks if using TG < 0.2 ng/mL as a functional sensitivity cutoff.

## Figures and Tables

**Table 1:** Baseline demographics.

Variable	Benign group *n* = 20	Cancer group *n* = 25	*p* value
Age (years)	52.9 (16.48)	50.7 (16.15)	0.65
Age			0.42
<45 years	5 (25%)	9 (36%)	
≥45 years	15 (75%)	16 (64%)	
Ethnicity			0.44
White	18 (90%)	21 (84%)	
Not White	2 (10%)	4 (16%)	
Gender (female)	15 (75%)	18 (72%)	0.82
Preoperative thyroid disease			0.42
Hyperthyroidism	4 (20%)	4 (16%)	
Hypothyroidism	3 (15%)	8 (32%)	
None	13 (65%)	13 (52%)	
Preoperative TG (ng/mL)			*0.0006* ^∗^
Median (quartiles)	167.5 (72.4, 598.5)	30.8 (8.9, 120.0)	
(Min, max)	(30.2, 2349.0)	(0.4, 264.0)	
Serum creatinine (mg/dL)			0.85
Median (quartiles)	0.8 (0.7, 0.9)	0.8 (0.7, 0.9)	
(Min, max)	(0.6, 1.1)	(0.6, 4.6)	
Histology			
Classical papillary (PTC)	—	15 (60%)	—
Follicular variant PTC	—	7 (28%)	
NIFTP	—	3 (12%)	
Stage			
I	—	16 (64%)	—
II	—	4 (16%)	
III	—	4 (16%)	
IV	—	1 (4%)	
RAI therapy (yes)	—	7 (31.8%)	—
ATA risk			—
High	—	1 (4%)	
Intermediate	—	8 (32%)	
Low	—	16 (64%)	
Response to therapy			—
Excellent	—	17 (68%)	
Indeterminate	—	7 (28%)	
Biochemically incomplete	—	1 (4%)	

## Data Availability

The clinical data used to support the findings of this study are included within the article.
